# Detection and monitoring of early dental caries and erosion using three-dimensional enhanced truncated-correlation photothermal coherence tomography imaging

**DOI:** 10.1117/1.JBO.26.4.046004

**Published:** 2021-04-29

**Authors:** Sohrab Roointan, Pantea Tavakolian, Koneswaran S. Sivagurunathan, Andreas Mandelis, Stephen H. Abrams

**Affiliations:** a University of Toronto, Center for Advanced Diffusion-Wave and Photoacoustic Technologies, Department of Mechanical and Industrial Engineering, Toronto, Ontario, Canada; b University of Toronto, Institute of Biomedical Engineering, Toronto, Ontario, Canada; c University of Toronto, Institute for Advanced Non-Destructive and Non-Invasive Diagnostic Technologies, Toronto, Ontario, Canada; d Quantum Dental Technologies, Toronto, Ontario, Canada

**Keywords:** active thermography, dental caries detection, dental erosion detection, dental thermal imaging, enhanced truncated-correlation photothermal coherence tomography, non-invasive thermophotonic imaging

## Abstract

**Significance:** Dental caries is the most common oral disease, with significant effects on healthcare systems and quality of life. Developing diagnostic methods for early caries detection is key to reducing this burden and enabling non-invasive treatment as opposed to the drill-and-fill approach.

**Aim:** The application of a thermophotonic-based 3D imaging modality [enhanced truncated-correlation photothermal coherence tomography (eTC-PCT)] to early dental caries is investigated. To this end, the detection threshold, sensitivity, and 3D lesion reconstruction capability of eTC-PCT in imaging artificially generated caries and surface erosion are evaluated.

**Approach:** eTC-PCT employs a diode laser with pulsed excitation, a mid-IR camera, and an in-house developed image reconstruction algorithm to produce depth-resolved 2D images and 3D reconstructions. Starting with healthy teeth, dental caries and surface erosion are simulated *in vitro* through application of specific demineralizing/eroding acidic solutions.

**Results:** eTC-PCT can detect artificial caries as early as 2 days after onset of artificial demineralization and after 45 s of surface erosion, with a laser power equivalent to 64% of maximum permissible exposure. In both cases, the lesion is not visible to the eye and undetected by x-rays. eTC-PCT is capable of monitoring lesion progression in 2-day increments and generating 3D tomographic reconstructions of the advancing lesion.

**Conclusions:** eTC-PCT shows great potential for further development as a dental imaging modality combining low detection threshold, high sensitivity to lesion progression, 3D reconstruction capability, and lack of ionizing radiation. These features enable early diagnosis and frequent monitoring, making eTC-PCT a promising technology for facilitating preventive dentistry.

## 1 Introduction

Dental caries is the most prevalent disease in humans.^1^^,^^2^ It is estimated that up to 90% of school-aged children and almost 100% of adults suffer from dental decay of varying severity at some point in their lives.^3^ Furthermore, the high consumption of sugar and acid-rich snacks and drinks in the modern western lifestyle can be traced to be the primary cause of dental demineralization and erosion,^4^ in particular for youth. The high prevalence of this disease also accounts for more than 5% of global health expenditures, and accounting for the indirect consequences of the disease (reduced productivity, lost working days, etc.), dental diseases resulted in global costs of US$442 billion in 2010.^3^ Due to this massive financial and health impact, the World Health Organization has put major emphasis on improvements to dental health, with one of the primary priorities being “development of oral health systems and orientation of services toward prevention and health promotion.”^3^ This implies that research and development toward technologies capable of early and accurate caries detection is highly desirable for the advancement of preventive dentistry methodologies.

Currently, visual inspection, followed by x-ray radiography is the primary dental diagnosis procedure in clinical use. However, while x-rays provide a visually accurate image of the tooth structure, they have limitations with respect to sensitivity to early caries and a poor record for locating lesions in regions other than the interproximal surfaces (i.e., occlusal surfaces and buccal or lingual surfaces that are perpendicular to the x-ray beam direction in clinical settings). Furthermore, x-rays are unsuitable for routine and frequent monitoring as they constitute potentially harmful ionizing radiation. Technologies based on the photothermal effect (the absorption of light in materials resulting in thermal wave generation) are one of the approaches through which researchers have attempted to address the limitations of radiography, with the PTR-LUM Canary System^5^^,^^6^ having been commercialized and showing excellent promise.^7^^,^^8^ However, as the Canary System represents a point measurement method, the next step would be to develop a thermophonic dental imaging modality.

Thermophotonic lock-in imaging [TPLI, an advancement on conventional lock-in thermography (LIT)] is a non-destructive active thermography evaluation modality based on the photothermal effect. It involves the detection of photothermal waves through emitted thermal infrared (IR) photons (Planck radiation) from tissues, captured by a mid-IR (MIR) camera. Thermophotonic images are spectrally resolved photothermal images from tissues with minimally elevated temperature due to optical absorption, with optical property contrast (absorption coefficient) amplified by superposed thermal property contrast (thermal diffusivity). TPLI has been successfully applied^9^^,^^10^ to dental imaging at 808 nm using a laser source. It has been reported^11^ that TPLI achieves superior sensitivity and specificity to the onset of mineral loss (demineralization) in early dental caries compared with spectral-domain cross-polarization optical coherence tomography (CP-OCT). As an active thermography technique, this modality uses a frequency-modulated light source (typically a laser) to induce heat inside a sample, while the evolution of temperature at the sample surface and in spectrally non-opaque subsurface regions of interest (ROI) is monitored by an IR camera. The temperature at each point on the surface is related to the subsurface features of the sample beneath that point, resulting in a depth-integrated 2D output image over the whole sample surface.

Because the thermal diffusion length is a function of the modulation frequency, LIT can interrogate deeper inside the sample through the use of lower modulation frequencies. However, optical source modulation at a constant frequency leads to depth-integrated results. Furthermore, the laser power level plays a key role in the output quality of a photothermal-based system, and for the results of *in-vitro* studies to be clinically relevant, they must be conducted at power levels that are lower than the clinically allowed maximum permissible exposure (MPE) for human soft tissue.^12^ Adherence to this limit is crucial because, for imaging methods such as LIT and TPLI, the laser beam size must cover the tooth surface, and in a clinical setting, this means that the patient’s gum line or other oral soft tissues might also be exposed to the laser.

Building on the high sensitivity of 808-nm excitation TPLI to the demineralization process of dental caries, we recently introduced the enhanced truncated-correlation photothermal coherence tomography (eTC-PCT) thermophotonics-based imaging technology, a depth-resolved 3D dental imaging modality.^13^ eTC-PCT has been applied to the imaging of naturally occurring, coarse dental defects, which demonstrated the capability of the technique to perform non-invasive 3D imaging of teeth.^13^ The eTC-PCT modality is MPE compatible and features a highly optimized algorithm for 3D reconstruction. Additionally, the capabilities of this technology for biomedical applications have also been successfully applied to early tumor imaging in small animals.^14^ eTC-PCT is an evolution of the original TC-PCT method^15^^,^^16^ and employs a highly optimized algorithm for 3D reconstruction.^17^ The eTC-PCT system output consists of consecutive 2D thermal images (slices), each corresponding to a different depth/signal delay. Compilation of slices with subsurface depth as a parameter results in 3D tomographic reconstruction. To increase the signal-to-noise ratio (SNR) of photothermal images, eTC-PCT uses a chirped pulse excitation with fixed pulse width, performs pulse-compression, and uses a time-evolving filter controlled by pulse delay and time slicing width.^17^

To assess this capability for translation into clinical use, the eTC-PCT detection threshold/sensitivity for early dental caries and surface erosion, as well as its capability for monitoring lesion progression, must be evaluated. The current study reports this evaluation accomplished through longitudinal imaging of controlled, artificial demineralization using established protocols on multiple healthy tooth samples. These images are also compared with the output of single frequency-thermal wave radar (SF-TWR) imaging,^18^ a more conventional depth-integrated 2D thermophonic imaging technology, which is also representative of the output achieved by LIT (see the Appendix). Furthermore, the 3D reconstruction capabilities of eTC-PCT for characterizing and visualizing the subsurface extent of early lesions were tested, including the demonstration of the ability of eTC-PCT to image early dental surface erosion.

## 2 Instrumentation and Methods

### 2.1 Dynamic Photothermal Imaging

**Fig. 1:**
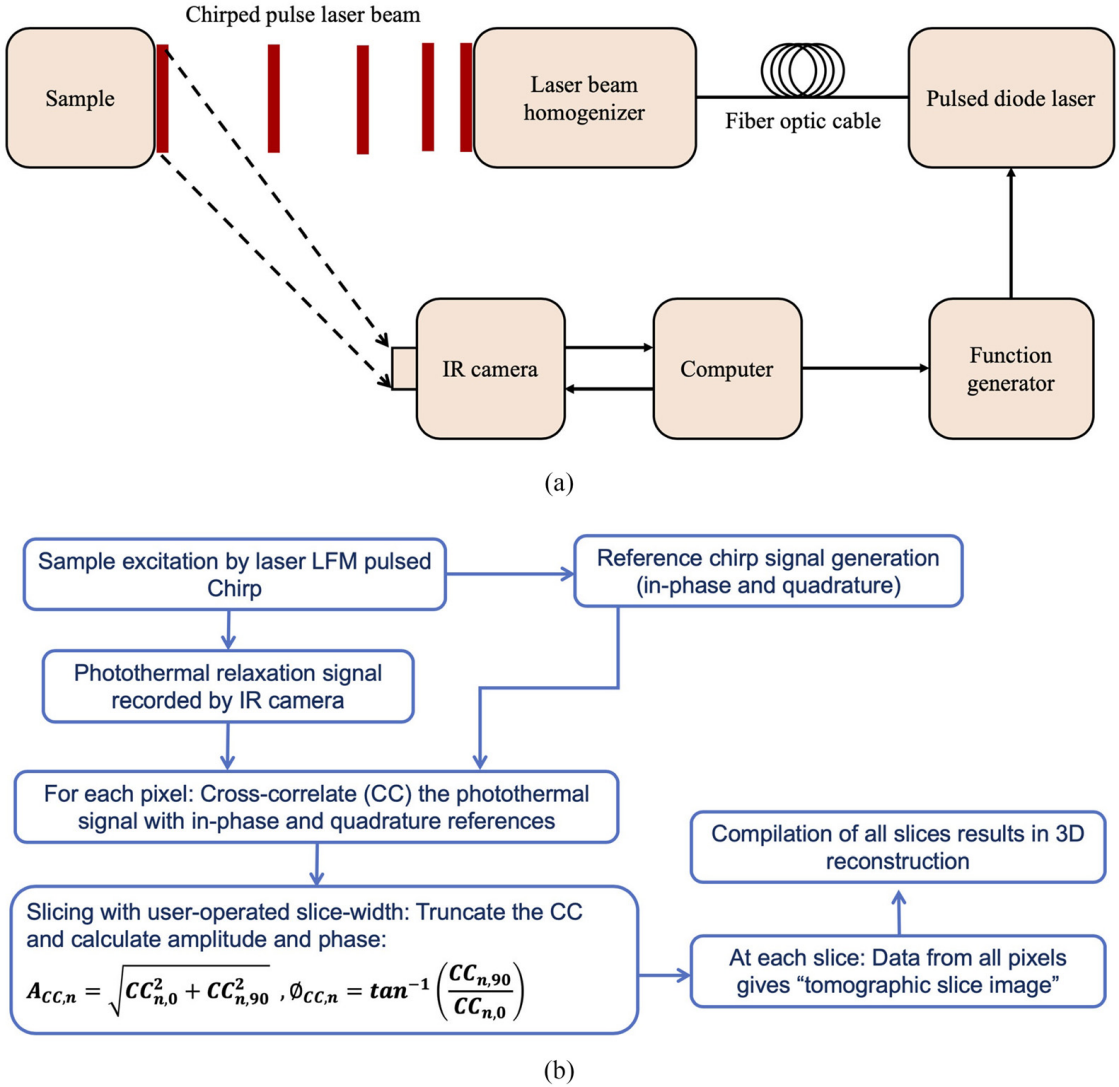
eTC-PCT modality: (a) eTC-PCT system schematic and (b) eTC-PCT simplified algorithm flowchart.

The dental imaging setup is shown in Fig. 1(a). The sample is placed on the imaging platform at the focal point of an MIR camera. The camera records the photothermal evolution of the sample following exposure to laser irradiation with an 808-nm laser. This study primarily used the eTC-PCT method and SF-TWR for comparison of 3D and 2D imaging, respectively. The latter was selected following a comparison (see the Appendix) between conventional LIT and SF-TWR dental imaging, which demonstrated that SF-TWR produced superior images at higher modulation frequencies, thereby representing the optimum dynamic thermographic modality in the context of the primary comparison with eTC-PCT in this study.

### 2.2 eTC-PCT Overview

**Fig. 2:**
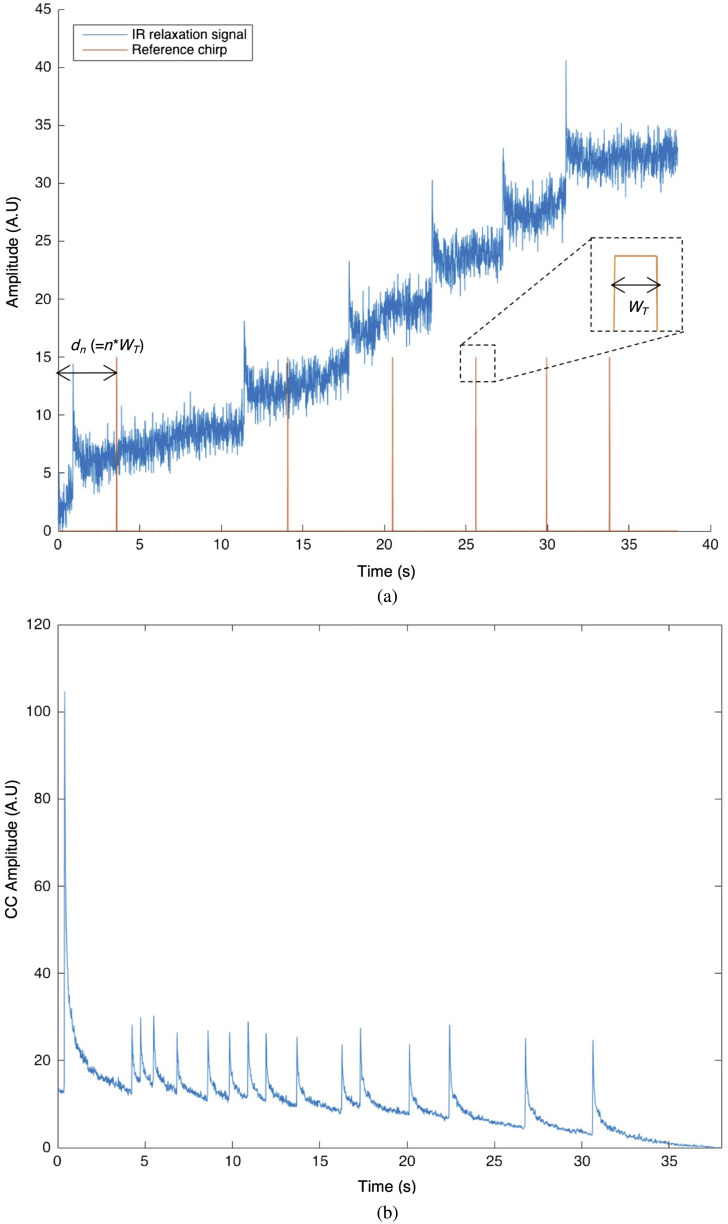
eTC-PCT slice calculation. (a) Typical raw thermal relaxation signal recorded by the IR camera for one pixel (in blue), and the corresponding synthesized reference chirp after delay unit $d_{n}$ (in orange). Slice width $W_{T}$ is selected by the operator and the delay unit $d_{n}$ is incremented as multiples n of $W_{T}$, where n is the slice number. Cross correlation is calculated at each $d_{n}$. (b) Typical CC result of the synthesized reference chirp signal and the IR thermal relaxation signal for one pixel at d=0 (i.e., the initial slice with n=0). Note the very narrow FWHM of the CC, the key feature of high axial resolution of eTC-PCT despite the diffusive nature of the thermal wave.

eTC-PCT uses linear frequency-modulated (LFM) chirp pulse laser irradiation, with the excitation pulse width, chirp frequency range, and chirp duration selected based on the desired detection depth and accuracy. The eTC-PCT algorithm is presented in Fig. 1(b). Following data acquisition, a reference chirp signal is generated (in-phase and quadrature) based on the excitation signal and subsequently, for each pixel of the image, the synthesized reference chirp is cross-correlated with the photothermal relaxation signal of that pixel recorded by the MIR camera. The cross-correlation (CC) evolves as the thermophotonic waves generated inside the sample reach the interrogated surface conductively, radiatively, or in combination.^15^^,^^17^ These thermal transients carry information about the morphology and optical and thermal properties of the layers beneath the surface, with information from deeper layers taking a longer time to arrive at the surface.^19^ The CC is truncated at consecutive time intervals by a time gating filter, which is calculated based on the “slice width,” $W_{T}$, and an “incremental delay unit,” d, where $W_{T}$ is controlled by the operator and is used for the calculation of d (Fig. 2). This filtering is employed for extracting depth-resolved information.^17^ Finally, for each d, compilation of the data from all pixels leads to a depth-resolved 2D “slice” image of the sample, and the compilation of 2D slices results in the 3D model of the sample. In this manner, for each pixel, three CC contrast channels are generated and used for the current study: amplitude peak, amplitude peak delay time, and phase.^17^

In eTC-PCT, the two amplitude-based channels correlate with the intensity of the thermal relaxation signal, while the phase channel correlates with the difference in arrival time of the thermal signal to the surface from different points inside the sample. Importantly, eTC-PCT axial resolution is determined by both the sample composition and $W_{T}$. A longer $W_{T}$ will compile data from more recorded frames for CC calculation of each pixel at each delay time and thus results in higher contrast and SNR (suitable for 2D slice images with high SNR), at the cost of reduced axial resolution. Conversely, the high axial resolution of a shorter $W_{T}$ is more suitable for 3D reconstruction.

### 2.3 SF-TWR Overview

**Fig. 3:**
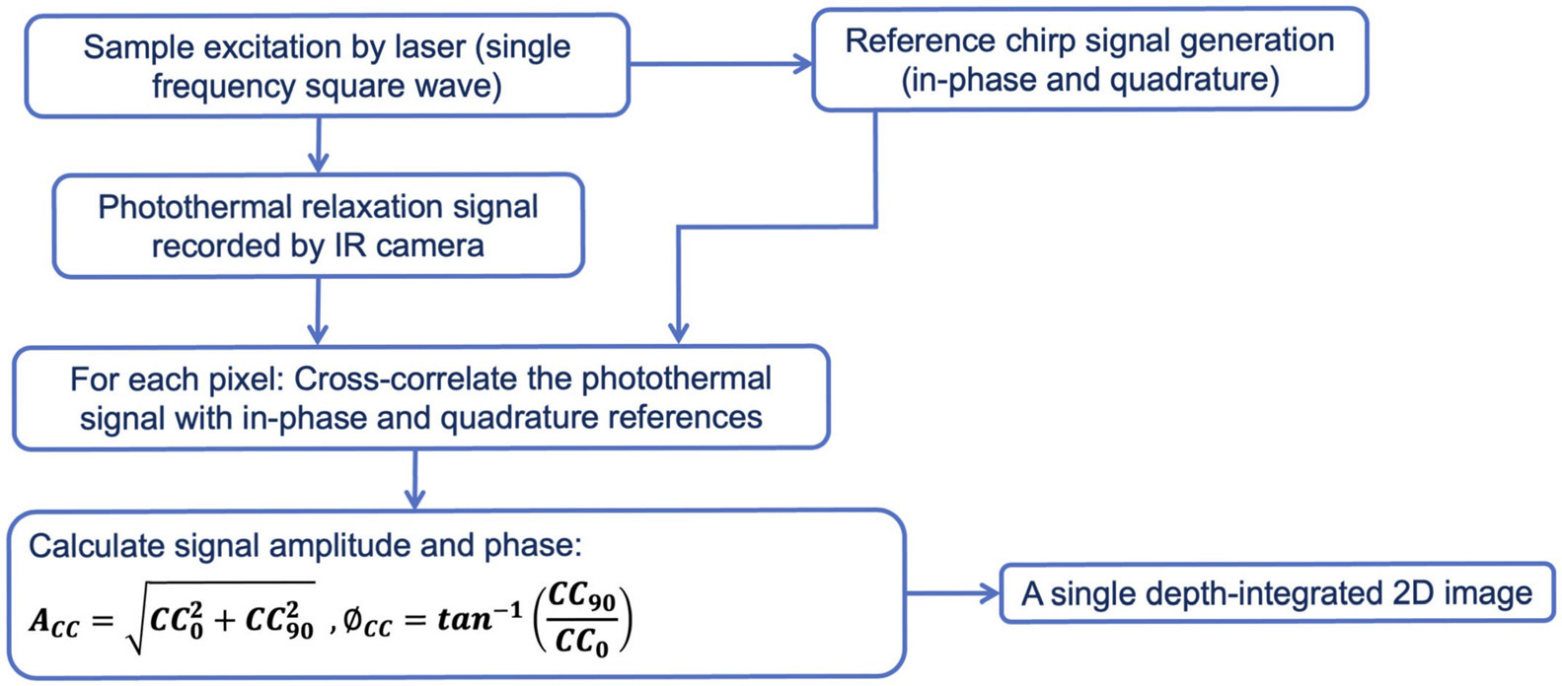
SF-TWR system. SF-TWR simplified algorithm flowchart.

The same imaging setup was used for SF-TWR imaging. This method uses a single-frequency square wave excitation signal for laser irradiation, with the modulation frequency and excitation duration selected based on the desired detection depth. In a manner similar to eTC-PCT, a reference chirp is calculated (in-phase and quadrature) based on the excitation signal. This reference chirp is then cross-correlated with the photothermal relaxation signal of each pixel. In contrast to eTC-PCT, no truncation algorithm is performed on the CC results, and the system output consists of a single depth-integrated 2D output image reconstructed using the amplitude and phase of the CC. The simplified algorithm is shown in Fig. 3. As reported previously^18^ and further explored in the Appendix, SF-TWR imaging contrast is similar to LIT at low modulation frequencies but significantly increases at higher frequencies with the added advantage of improved spatial resolution.

### 2.4 Optimized Dental Imaging Parameters

The optimal parameters for dental imaging using eTC-PCT and SF-TWR systems were determined based on experimental constraints and trial and error. For eTC-PCT, the primary constraints were minimizing imaging duration and laser exposure, while retaining sufficient SNR for 3D reconstruction. To compare SF-TWR with eTC-PCT, the excitation duration for the former method was chosen to be the same as eTC-PCT, while using the maximum allowable laser power (i.e., equal to MPE) and an optimized modulation frequency. The parameters are presented in Tables 1 and 2.

**Table 1:** eTC-PCT optimized parameters.

Method	Excitation wave form	Excitation duration	Chirp frequency range	Pulse width	Energy density per pulse	Slice width ($W_{T}$)
eTC-PCT	Pulsed LFM chirp	12 s	0.2 to 0.6 Hz	40 ms (total of five pulses in 12 s)	$0.54\;J/{\mathrm{cm}}^{2}$ (64% of MPE, ∼24 mm incident beam diameter)	80 ms for 2D slice outputs
						20 ms for 3D reconstruction

**Table 2:** SF-TWR optimized parameters.

Method	Excitation wave form	Excitation duration	Modulation frequency	Power density
SF-TWR	Single-frequency square wave	12 s	0.3 Hz	$0.3\;W/{\mathrm{cm}}^{2}$ (100% MPE, ∼24 mm incident beam diameter)

### 2.5 Tooth Selection and Experimental Protocols

In compliance with the research ethics guidelines of the University of Toronto, a large number of extracted teeth from anonymous donors were collected from local dental offices. After choosing a few test candidate samples, visual assessment and ranking was performed by a practicing dentist using ICDAS II (International Caries Detection and Assessment System) criteria,^20^ and only healthy molars with no visible defects (ICDAS II score of 0) were chosen for this study. The samples were not modified in any way, other than the complete removal of soft tissue. The samples were then mounted on LEGO bricks using epoxy and stored in distilled water inside a refrigerator. All samples were imaged with our eTC-PCT and SF-TWR instrumentation systems in their healthy state as a reference before any modification.

### 2.6 Artificial Demineralization Protocol

The artificial demineralization solution used for this study consisted of a mix of 0.1 M lactic acid and 0.1 M NaOH, at a pH level of 4.5, which is a well-established protocol,^10^^,^^21^^,^^22^ leading to the inception of a lesion just below a thin, healthy surface layer of the tooth, which is the hallmark mineral profile of early dental caries. For the undertaken longitudinal study, the following steps were taken.

1. Each tooth sample was taken out of distilled water and air dried for 30 min.
2. The sample was covered with acid-resistant transparent nail polish except for a rectangular window on one of its surfaces, which was to be exposed to the artificial demineralization solution (“treatment window”). This procedure protects the rest of the tooth from the effects of the acid, creating a localized early lesion. The nail polish was then allowed to dry in air for 45 min, to ensure it would not diffuse over the window once inside the liquid acid.
3. Approximately 5 ml of demineralization solution were added to a falcon tube, and the sample was placed inside the tube, immersed in the solution. Care was taken so that the solution did not contact the LEGO brick or the epoxy. The tube containing the sample was kept in an enclosed container with a water reservoir to preserve the humidity level of the tooth.
4. After 48 h, the sample was taken out of the solution and rinsed for 1 min in tap water to wash off the acid. The nail polish was then removed with acetone, and the sample was rinsed again for 5 min to ensure that no acetone/nail polish residue was left on the sample. The sample was then stored in distilled water for 24 h to rehydrate.
5. The sample was taken out of the distilled water and dried with forced air for 3 min and then dried in air for 20 min. The surface of the sample with the treatment window was then imaged using the photothermal imaging systems.
6. After imaging, steps 1 to 5 were repeated again so that the sample was imaged after each 48-h period of increasing demineralization of the designated treatment window. This procedure was repeated for up to a total of 10 days (240 h) of acid exposure, resulting in later stages of carious lesion.

## 3 Smooth Surface Caries Imaging

### 3.1 eTC-PCT Early Demineralization Imaging

**Fig. 4:**
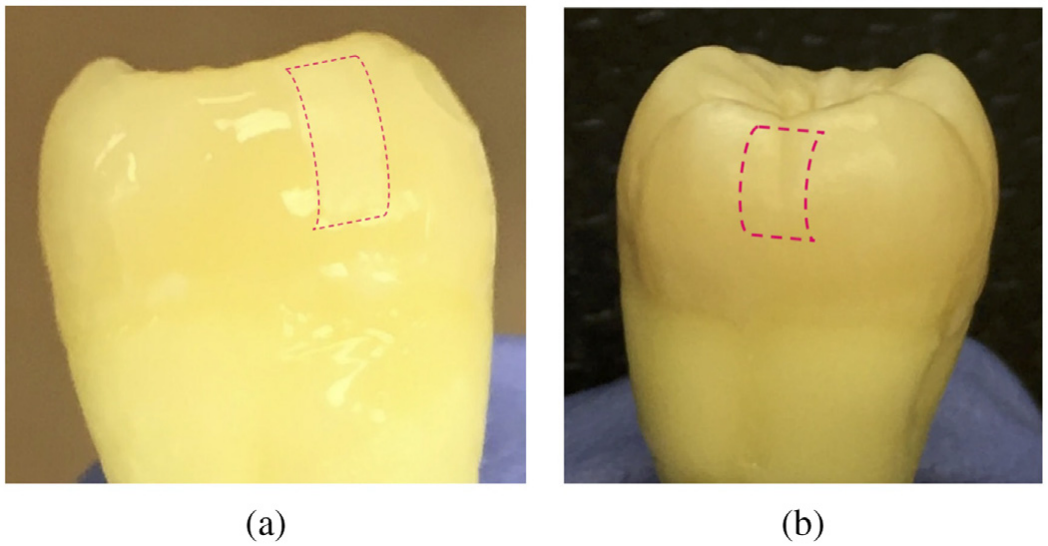
Samples D1 (a) and D2 (b). The digitally added pink outlines designate the treatment window on each sample.

Two extracted human molar teeth (henceforth referred to as D1 and D2) were used in this study (Fig. 4), with their designated treatment windows digitally marked with a pink outline in the image. Following the established artificial caries-generating demineralization protocol described in Sec. 2.6, samples D1 and D2 were exposed to acid for 48-h intervals and a total of 10 days each. The appearance of the teeth in visible light did not change after this time, suggesting that the resulting caries was still in its early stages.

**Fig. 5:**
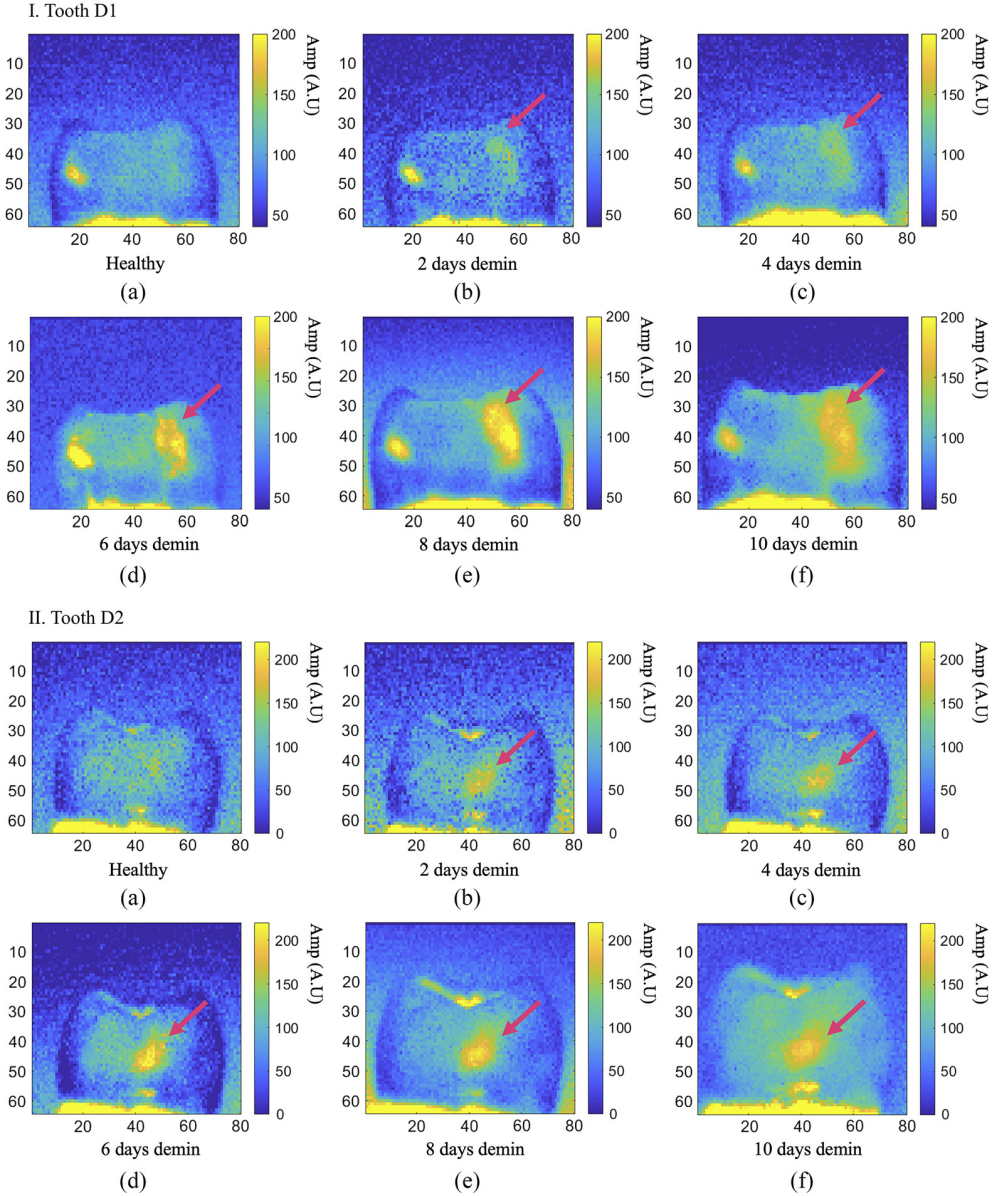
Longitudinal demineralization results from the eTC-PCT amplitude channel. Each image is the first 2D slice of the eTC-PCT output, containing the near-surface data. Part I (a)–(f) and part II (a)–(f) The evolution of eTC-PCT amplitude results for D1 and D2, respectively, from a healthy state to having had the treatment windows exposed to the artificial demineralization solution for 10 days, creating growing caries (marked with the pink arrow). The caries signal intensity is directly related to its severity. However, after day 6, the amplitude data become saturated and the images cannot reliably show the progression of the lesion. This may be due to the well-known photothermal saturation effect that occurs at high absorption coefficient values. The highly absorbing regions on the left and bottom of the tooth surface are due to a pre-existing lesion and the cementum, respectively. Dimensions for all images: W=1.25  cm (±0.1), H=1  cm (±0.1).

The eTC-PCT amplitude images taken from D1 and D2 are presented in parts I and II of Fig. 5, respectively. The treatment window (and the resulting caries) is marked with a red arrow. The images show the first eTC-PCT 2D amplitude slice from each experiment, which contains the near-surface data. In both samples, tooth cementum is visible at the bottom of the images as a highly absorbing region. For sample D1, the highly absorbing region on the left side of the tooth is a pre-existing demineralization. The amplitude images from each imaging session have been normalized with respect to the pixel values of the healthy region of the tooth from that particular experiment. In these images, it can be seen clearly that the eTC-PCT amplitude channel is capable of detecting the changes as early as 2 days after onset of demineralization, demonstrating its detection sensitivity threshold. The results clearly show the ability of this modality to monitor the progress of the caries from the healthy state to 6 days of demineralization, which is a measure of its sensitivity to minute changes. However, the results from day 6 to day 10 show that the amplitude channel becomes gradually saturated and less sensitive to change in caries severity.

**Fig. 6:**
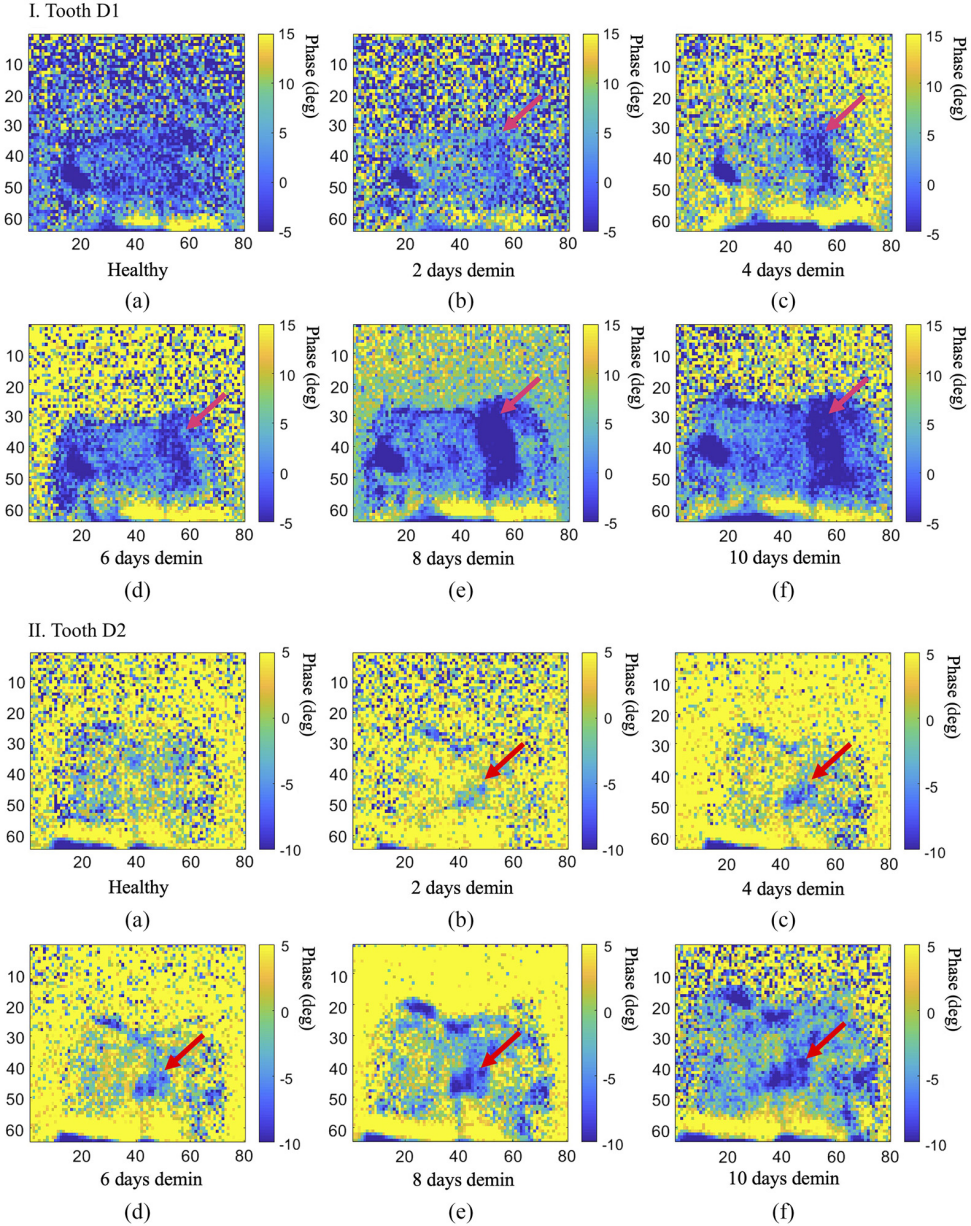
Longitudinal demineralization results from the eTC-PCT phase channel. Each image is the first 2D slice of the eTC-PCT output, containing the near-surface data. Part I (a)–(f) and part II (a)–(f) The evolution of eTC-PCT phase results for D1 and D2, respectively, from a healthy state to having had the treatment window exposed to the artificial demineralization solution for 10 days, creating growing caries (marked with the pink arrow). The phase output exhibits an opposite trend to that of the amplitude, with caries exhibiting a lower phase value than healthy enamel, due to near-surface absorption of photons. Dimensions for all images: W=1.25  cm (±0.1), H=1  cm (±0.1).

**Fig. 7:**
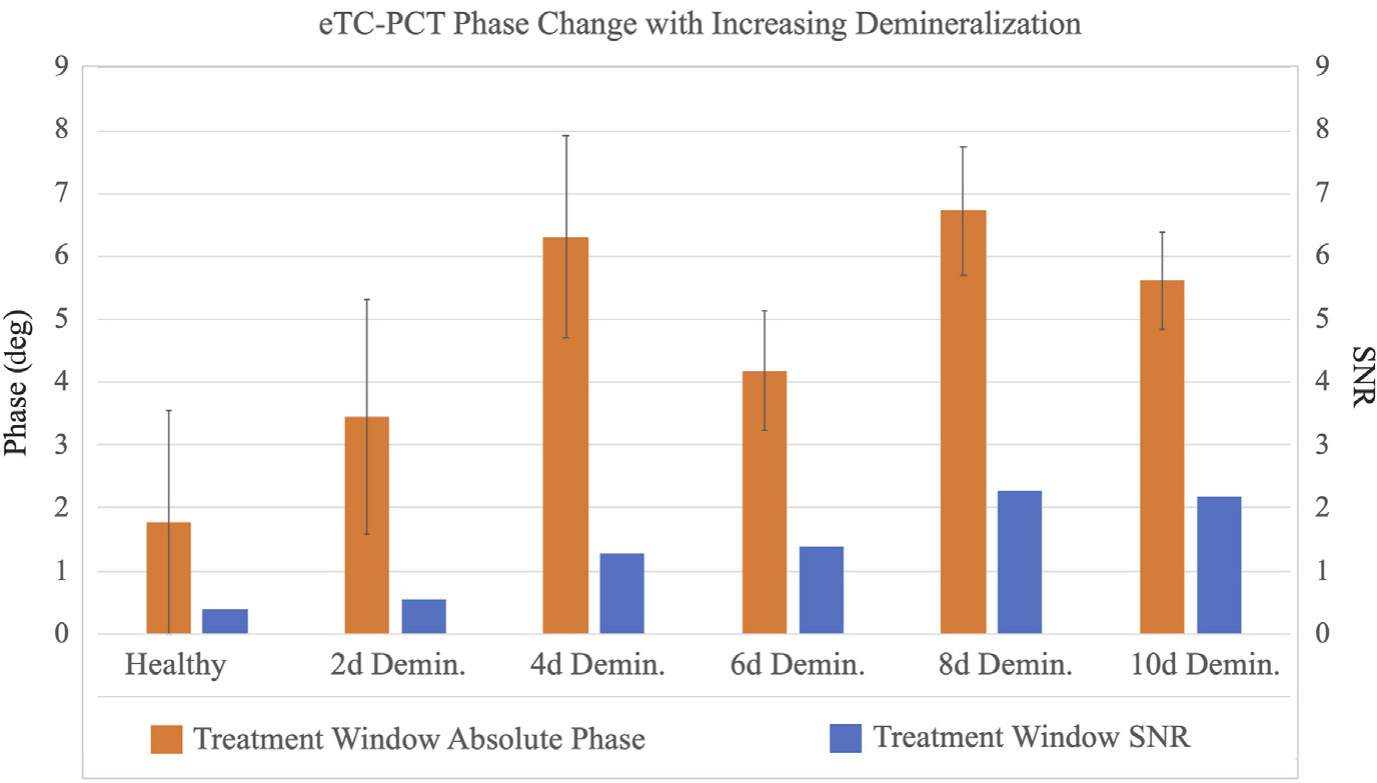
Quantification of eTC-PCT phase data from the treatment window on sample D1. While a generally positive trend is observed in the absolute value of the phase difference between the untreated and the increasingly demineralized treatment window, this value is prone to day-to-day experimental fluctuations. The treatment window SNR, however, shows a much more consistent trend and is less affected by experimental condition variations, making it a reliable indicator of caries progression. The first column from the left presents the treatment window phase data before any acid exposure, which show a high level of standard deviation (e.g., noise), as is typical of healthy enamel signals. Note that the plot presents the “absolute value” of the phase difference for better visualization. The actual phase of the demineralized region is lower than the healthy region.

The results from phase imaging for D1 and D2 are presented in parts I and II of Fig. 6, where each image presents the first eTC-PCT 2D phase slice from that experiment, which contains the near-surface data. It can be seen that the details in the image are much less pronounced compared with the amplitude channel, which is due to the phase channel containing only conductive photothermal information. While the phase channel shows more changes in contrast for the caries as the lesion progresses, due to day-to-day experimental changes, the absolute phase value of the pixels cannot be a reliable measure of lesion progression, as demonstrated in Fig. 7. However, the data in this figure show that, despite the fluctuations in pixel contrast, the increase in SNR and decrease in standard deviation (STD) of the pixels inside the treatment window exhibit a more consistent relationship with caries progression, thereby making these two factors highly indicative of the sensitivity of eTC-PCT to caries progression. The STD values are seen as error bars in Fig. 7. For SNR calculation, the signals from the treatment window and the healthy region were normalized, and the SNR of the treatment window is defined as

$$\mathrm{SNR}=\frac{M_{\text{net}}}{{\mathrm{STD}}_{H}},$$ (1)

where $M_{\text{net}}$ is the normalized mean (i.e., difference of means between the healthy region and treatment window) and ${\mathrm{STD}}_{H}$ is the standard deviation of the healthy region. The standard deviation of the normalized treatment window (${\mathrm{STD}}_{D}$) is defined as the STD of the difference between the mean phase value of the healthy region and the mean phase value of the treatment window,

$${\mathrm{STD}}_{D}=\sqrt{\frac{{\mathrm{STD}}_{H}^{2}}{n_{H}}+\frac{{\mathrm{STD}}_{C}^{2}}{n_{C}},}$$ (2)

where ${\mathrm{STD}}_{H}$ and ${\mathrm{STD}}_{C}$ are the standard deviations of the healthy region and treatment window, respectively, and $n_{H}$ and $n_{C}$ refer to the mean phase values of the same regions, respectively.

### 3.2 eTC-PCT 3D Tomographic Imaging of Early Demineralization

**Fig. 8:**
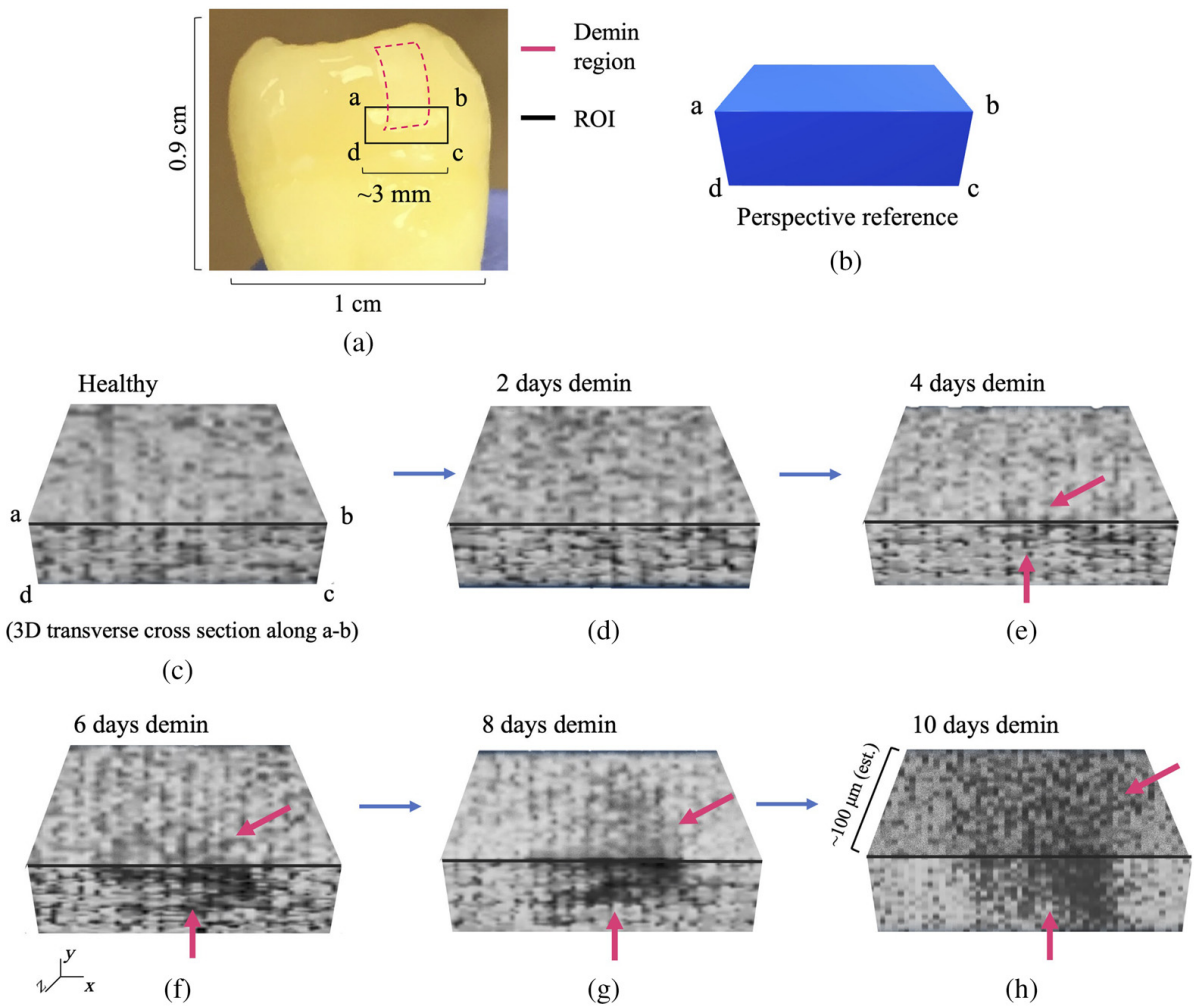
eTC-PCT 3D phase reconstruction of caries progression in sample D1. (a) Marked demineralization treatment region (pink outline) and the ROI on the tooth surface, the dental tissue behind which was reconstructed in 3D using the depth profilometry capability of eTC-PCT. (b) 3D perspective for the eTC-PCT 3D reconstructions. The intact (healthy) state of the treatment window is shown in 3D in (c), where the same ROI as marked in (a) is the front surface of the right parallelepiped cross-section, and the top surface shows the hidden “inside” of the tooth behind this surface ROI, as if the tooth has been cut along line a-b. (d)–(h) eTC-PCT 3D phase reconstructions of the progression of the artificial carious lesion for up to 10 days of acid exposure. The demineralized carious range, a progressively spreading dark region, is marked with pink arrows. In all 3D figures, the subsurface layers along the z-axis of the image are not on the same scale as the x-y plane. The scaling was performed to provide better visibility for the subsurface layers. These images demonstrate the tomographic capability of the eTC-PCT phase channel to detect caries as early as 4 days after the onset of treatment and to monitor its progression inside the tooth with high sensitivity.

**Fig. 9:**
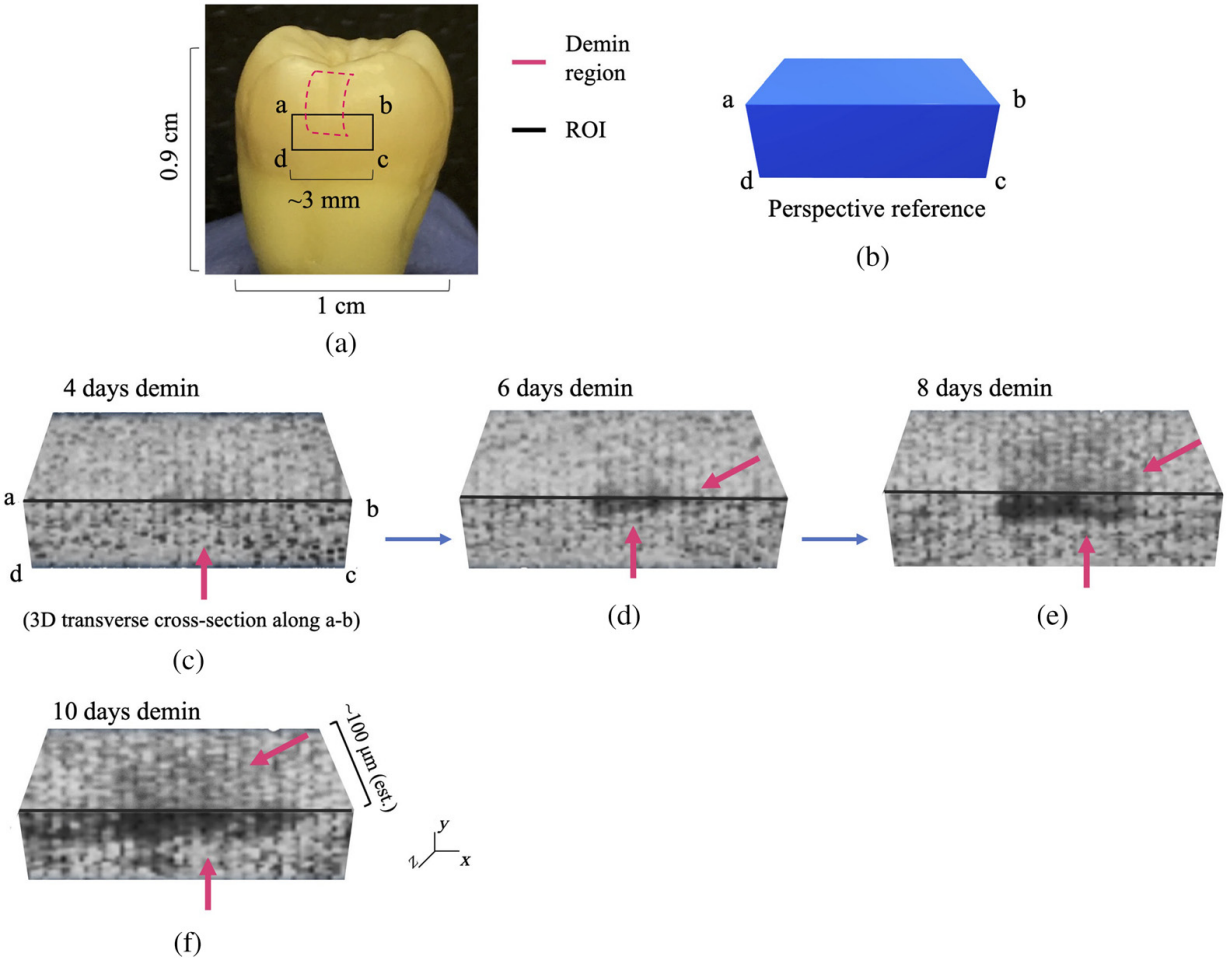
eTC-PCT 3D phase reconstruction of caries progression in sample D2. Similar to Fig. 8, (a) shows the marked demineralization treatment region (pink outline) and the ROI on the tooth surface. (b) The perspective reference for the 3D images. (c)–(f) The progression of caries inside the tooth along line a-b, behind the ROI surface. The results present the eTC-PCT 3D phase reconstructions of the lesion for up to 10 days of acid exposure. The demineralized carious region is marked with pink arrows and is the progressively spreading dark region on the tooth surface and inside the tooth enamel. In all 3D figures, the subsurface layers on the z-axis of the image are not on the same scale as the x-y plane. The scaling was performed to provide better visibility for the subsurface layers.

The sensitivity of the eTC-PCT phase channel combined with its tomographic capacity is the primary defining feature of the eTC-PCT imaging in producing 3D reconstructions of subsurface lesions. The phase data from all slices are used to create a 3D representation of the carious lesion, as presented in Figs. 8 and 9 for samples D1 and D2, respectively. Figures 8(a) and 9(a) show the reference markings for creating the 3D reconstructions, bordered by the rectangle marked by a, b, c, and d and a transverse cross-section of the tooth, virtually cut along line a, b which shows the “inside” of the tooth and the extent of the lesion within it. The images have been converted to grayscale for better visibility, and the darker pixels indicate lower phase values inside the carious region.

As can be seen in these figures, the 3D phase reconstruction clearly shows the extent of progressing caries inside the tooth at each 48-h interval, thus providing a reliable and sensitive visual reference for monitoring the lesion progress. It must be noted that, while the lesion depth can be estimated to be in the ∼100-μm range (based on other studies using the same demineralization protocol^23^^,^^24^), further studies must be performed to enable quantitative interpretation of the lesion depth based on eTC-PCT 3D outputs.

### 3.3 SF-TWR Early Demineralization Imaging

SF-TWR imaging was carried out on sample D1 and employed the same IR camera and 808-nm laser used in the eTC-PCT setup. The experiments were performed in parallel with eTC-PCT on tooth D1 at 6, 8, and 10 days of demineralizing the treatment window, using square waveforms and at laser modulation frequencies of 0.3, 0.5, 1, 2, and 5 Hz. The remaining imaging parameters in this study are described in Table 2. Maximum SNR for caries detection was achieved at 0.3 Hz. At the selected power level ($0.3\;W/{\mathrm{cm}}^{2}$, or 100% MPE) for frequencies above 5 Hz, the output became highly noised and did not provide any discernible contrast data from the tooth. However, it must be noted that, with power increased above the MPE compatibility level, SF-TWR (and by extension LIT) is capable of providing much more detail and contrast data at higher modulation frequencies, as was the case in a previous dental LIT (TPLI) study.^11^

**Fig. 10:**
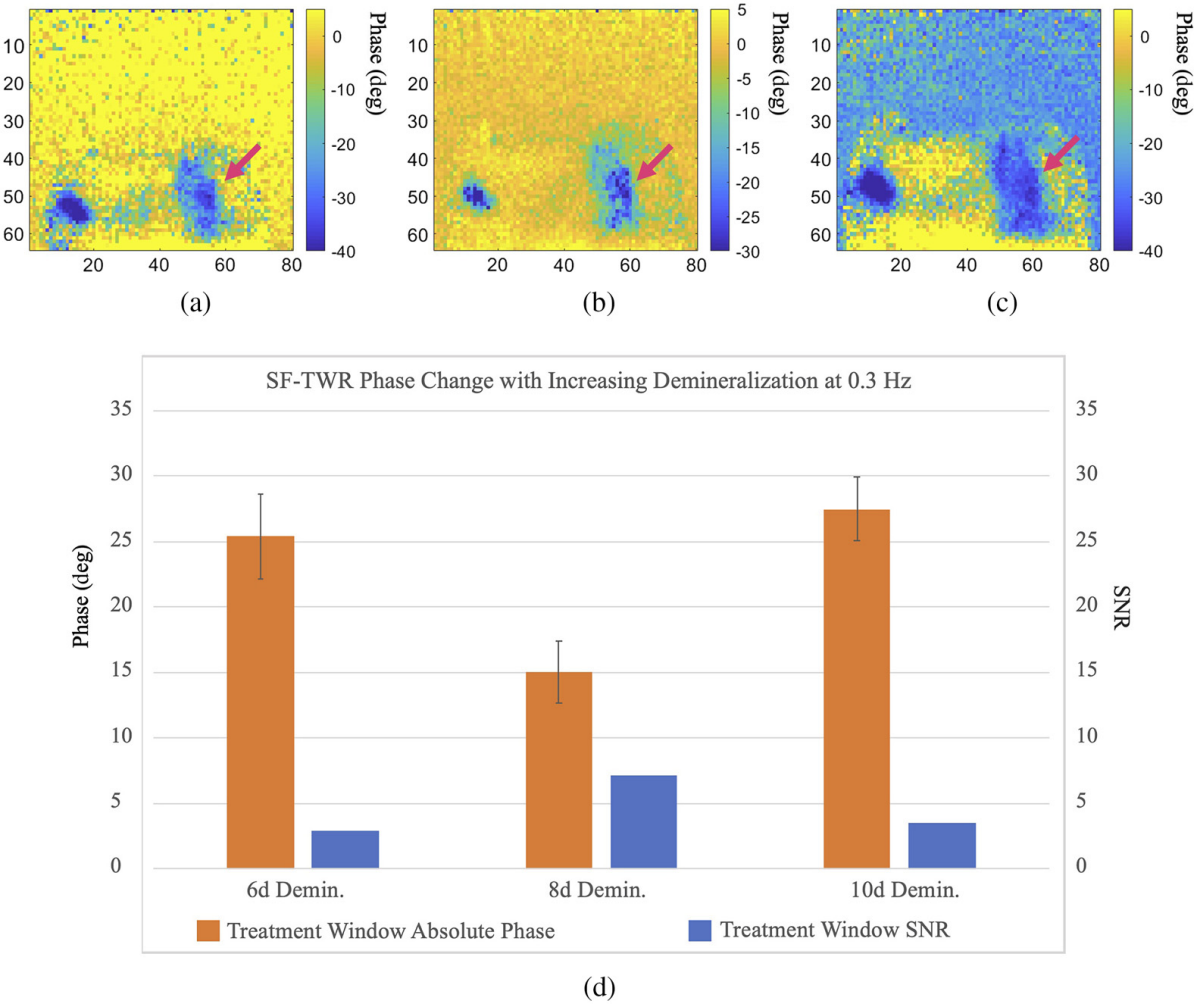
The 0.3-Hz SF-TWR longitudinal early caries imaging. (a)–(c) The SF-TWR phase images of sample D1 for early caries progression from 6 days to 10 days of demineralization. (d) The statistical data extracted from each image. While SF-TWR has high sensitivity for early caries with high SNR and contrast, it does not provide a reliable indicator of lesion progress. Dimensions for all images: W=1.25  cm (±0.1), H=1  cm (±0.1).

The results from SF-TWR at 0.3-Hz modulation frequency can be reasonably used for comparison with the eTC-PCT results presented in Figs. 6 and 7. To this end, Fig. 10 shows the phase output of 0.3-Hz SF-TWR imaging of artificially generated early caries at 6, 8, and 10 days of treatment window demineralization. The SNR and the STD (error bars) were calculated as described by Eqs. (1) and (2). It can be seen that SF-TWR is capable of providing high contrast and detail for the demineralized treatment window for all three acid exposure durations. Furthermore, for these three experiments, the SNR values at 0.3 Hz are generally higher than those achieved by eTC-PCT (Fig. 7). This difference stems from the depth-integrated nature of SF-TWR, as well as its use of square-waves compared with the low duty cycle quasi-pulses used in eTC-PCT. These results suggest that low modulation frequency SF-TWR (and by extension LIT) could be used effectively at MPE-compatible power levels for 2D detection and projectional imaging of early dental caries.

## 4 Surface Erosion Imaging

Surface erosion in teeth is a result of direct exposure of a tooth to acid (such as drinking orange juice), which results in removal of material without bacterial origin.^25^ eTC-PCT was applied to imaging this lesion type to further investigate its potential for dental erosion imaging. As opposed to demineralization caries, which at early stages presents itself as demineralization below a healthy surface enamel layer, erosion occurs as mass loss at the surface level with a secondary effect being the spread of demineralization in the layers immediately adjacent to the surface. As erosion progresses, its effect becomes visible on tooth surface as a chalky white region. For the present longitudinal surface erosion imaging study, the following protocol was employed using 35% phosphoric acid gel.

1. A tooth sample was taken out of distilled water and dried in air for 30 min.
2. Using a syringe, approximately 2-ml drop of the phosphoric acid gel was placed on a circular treatment window on the tooth.
3. After 15 s, the gel was washed off the tooth with water, and the tooth was rinsed for 5 min.
4. The tooth was then placed back into distilled water for 24 h. This was done to allow time for the secondary demineralization effect to occur beneath the eroded surface.
5. After 24 h, the tooth was taken out of distilled water, dried with forced air for 5 min, air-dried for 30 min, and imaged with the eTC-PCT system.
6. The above steps were repeated multiple times for increasing durations of phosphoric acid application in the following order: 15 s, +30  s (total acid exposure of 45 s), and +75  s (total acid exposure of 2 min).

**Fig. 11:**
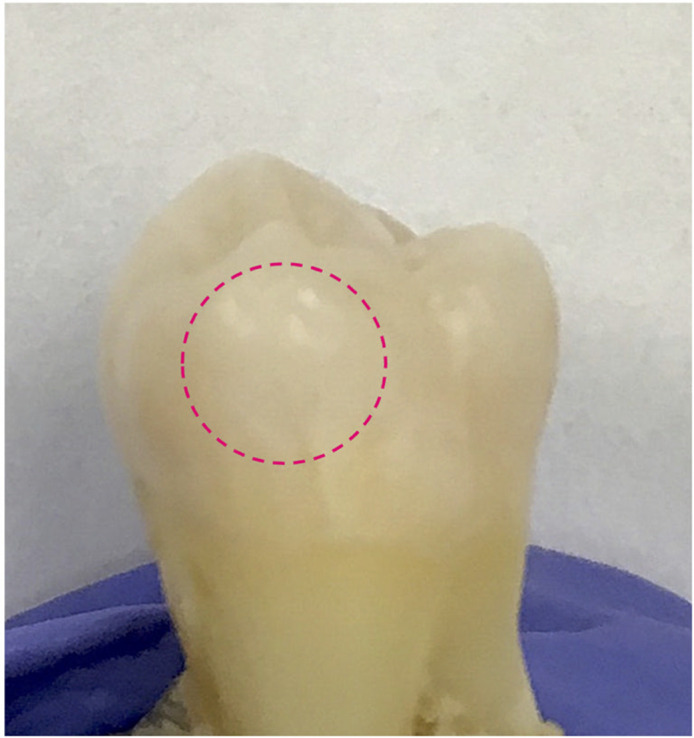
Visible light image of healthy tooth sample used for the dental surface erosion study. The surface of sample E1 used for this study shown here was assessed by a practicing dentist as healthy (ICDAS II score of 0). The image shows the tooth surface after 2 min of artificial erosion with the treatment region marked inside the pink circle.

**Fig. 12:**
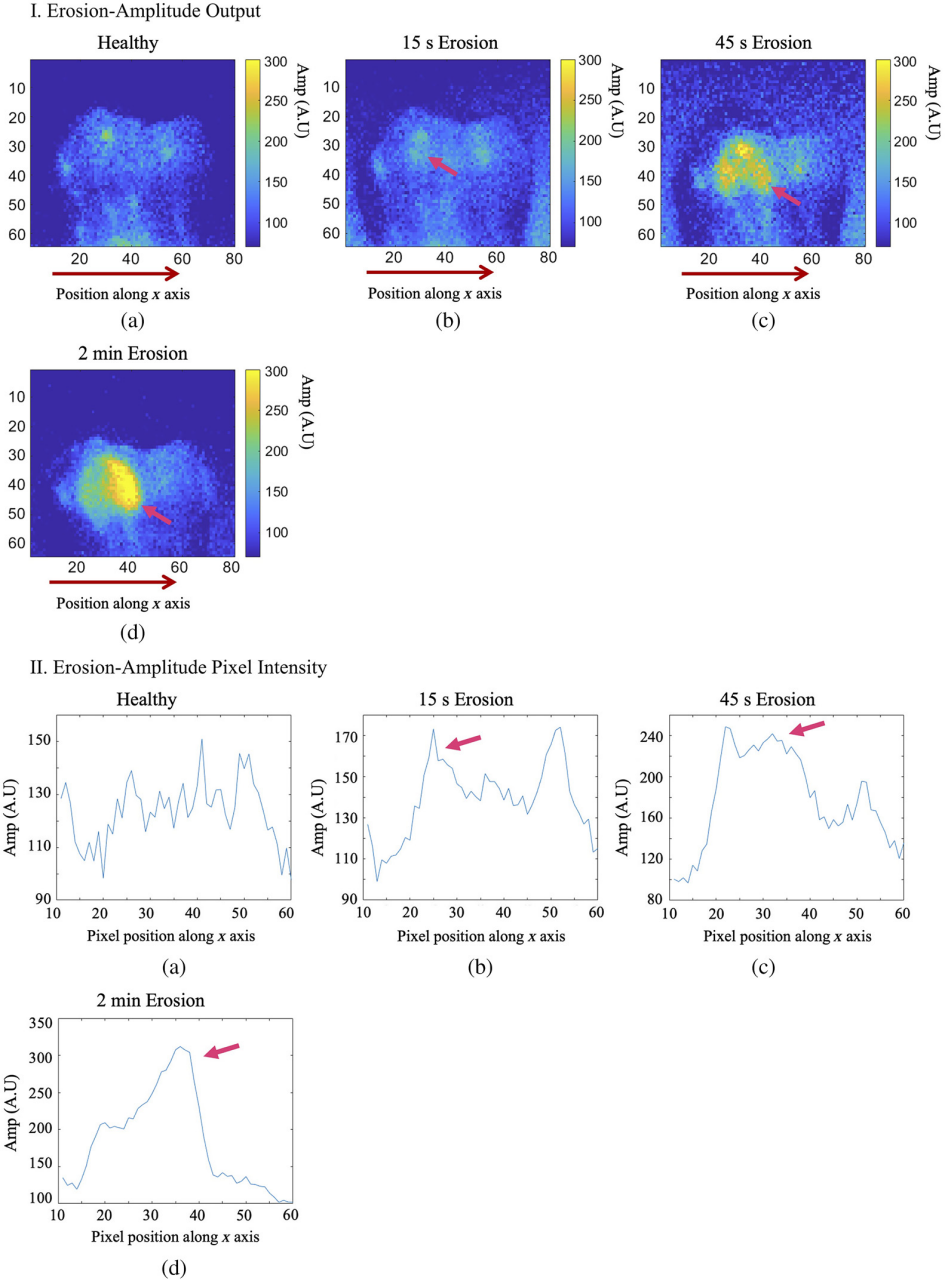
eTC-PCT amplitude channel results for imaging of artificial dental surface erosion on sample E1. Parts I and II present the imaging results and pixel intensity plots, respectively. The pixel intensity plots average the intensity of the pixels located on the tooth along the x-axis positions 10 to 60, also marked by the red arrow below each image. The signal from the eroded region is marked with pink arrows in these images. The amplitude channel provides relatively high sensitivity and SNR to dental erosion. Dimensions for all images: W=1.25  cm (±0.1), H=1  cm (±0.1).

**Fig. 13:**
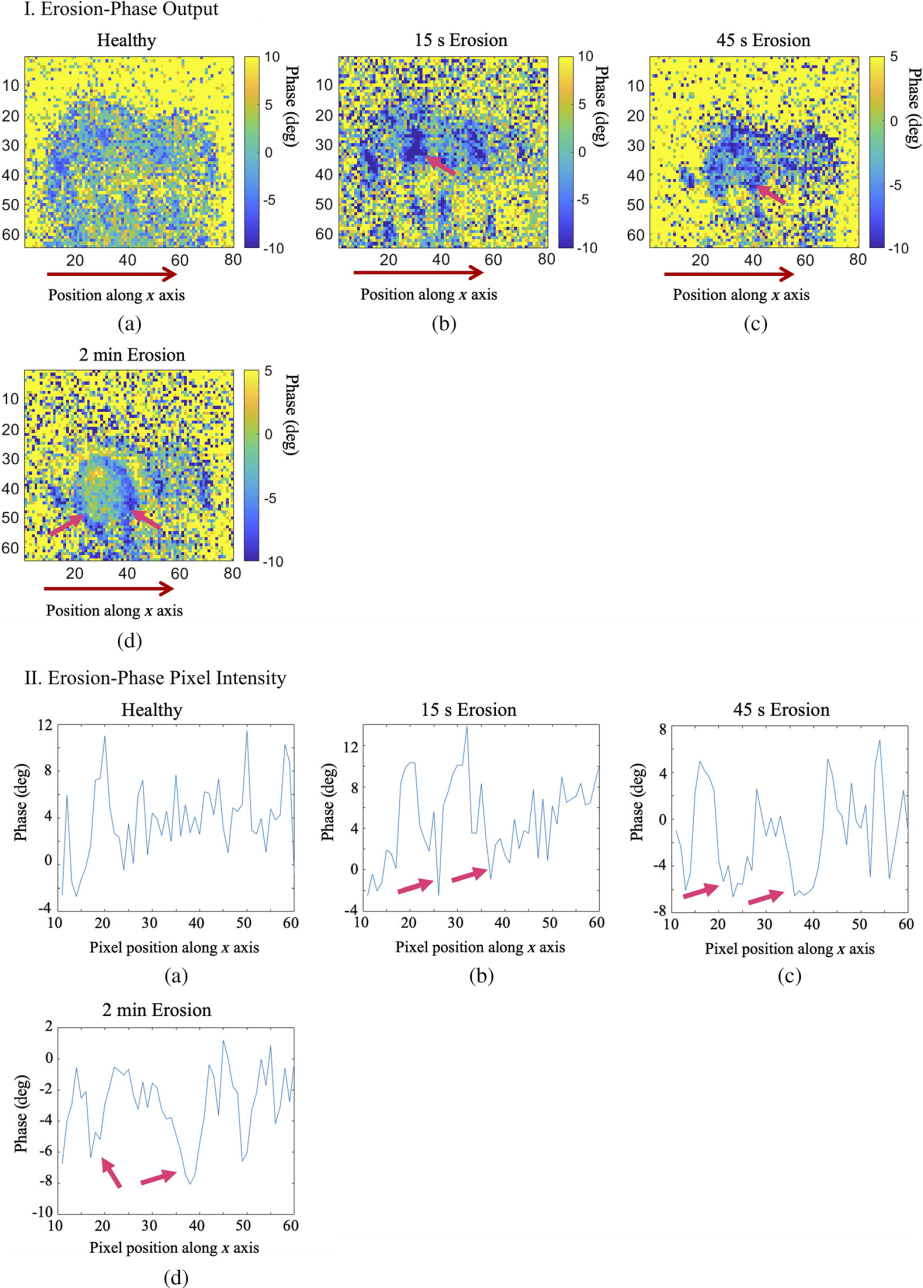
eTC-PCT phase channel results for imaging of artificial dental surface erosion on sample E1. Parts I and II present the imaging results and pixel intensity plots, respectively. The pixel intensity plots average the intensity of the pixels located on the tooth along the x-axis positions 10 to 60, also marked by the red arrow below each image. The signal from the eroded region is marked with pink arrows in these images. Compared with the amplitude channel, the phase channel provides lower sensitivity to change and lower SNR for dental erosion. Dimensions for all images: W=1.25  cm (±0.1), H=1  cm (±0.1).

Following the foregoing surface erosion protocol, tooth sample E1 was eroded for a total of 2 min on a specified treatment window, as digitally marked in Fig. 11. The treatment window was eroded and imaged at 3 steps of phosphoric acid gel application: 15 s, 45 s, and 2 min. The first (surface) image slices from the eTC-PCT imaging amplitude and phase results for sample E1 are presented in Figs. 12 and 13. In both figures, part I shows the amplitude and phase slice images, respectively, and part II presents the mean pixel intensity plots for each image. The pixel intensity plots are calculated by averaging the pixel amplitude/phase values on the tooth surface at locations along the red arrow below each image, thus providing a quantified interpretation of each image data. The pink arrow in each image marks the location of the applied erosion.

As can be seen in these results, the amplitude channel shows much higher contrast and sensitivity (i.e. signal change with increasing erosion) to surface erosion, while the phase data are highly noised. Nevertheless, the erosion becomes clearly visible in the eTC-PCT amplitude output after 45-s exposure to erosive conditions and after 15 s in the phase image. At this stage, no sign of erosion can yet be seen in visible light (Fig. 11). It is noted that the erosion does not appear to occur in a uniform manner across the treated region. This effect can be seen in Fig. 12, part I (d), where the right half of the eroded window generates a much stronger amplitude signal. The same effect is observed in the phase results, where erosion appears to be stronger in the outer region of the same section of the treated window. This is clearly observed in Fig. 13, part I (d) and part II (b)–(d), where two pink arrows in each image mark the increased erosion in the outer region of the treated window.

## 5 Discussion

In this study, the application of eTC-PCT, a new thermophotonic 3D imaging modality with improvements over the original TC-PCT technique,^17^ to dental caries imaging was demonstrated and was compared with SF-TWR (and, by extension, with LIT), which can generate 2D dental imaging. Importantly, it was demonstrated that eTC-PCT imaging is capable of detection and tomographic reconstruction of very early lesions through truncation of the thermophotonic temporal response signal into depth-resolved CC slices. It was confirmed that these early lesions were undetectable through visual inspection as well as through attempted micro-computed tomography (μCT) imaging. The amplitude- and phase-based eTC-PCT images can potentially be employed in detecting and monitoring early caries with a low detection threshold and high sensitivity to minute carious demineralization changes. As shown in these results, consistent with our previous images of natural dental defects,^13^ healthy enamel features a low amplitude signal and highly noised phase data due to the low photon absorption. For very early demineralization phases (up to 4 days), the higher contrast of the amplitude channel between intact and early carious regions makes it more useful than the considerably noisier phase channel. However, the results from day 6 to day 10 show that the amplitude channel becomes gradually saturated and less sensitive to change in caries severity. This problem is also present in results with other modalities such as CP-OCT.^11^ It is worth noting that even minor day-to-day differences between laser intensity or the positioning of the tooth (e.g., distance to laser) can have considerable influence on the amplitude channel output, thus making it less reliable than phase for fine monitoring of lesion progress, especially at more advanced stages of caries. Another complicating factor is that the repeated application and removal of nail polish on the tooth surface might alter its emissivity, which affects the amplitude channel due to the signal dependence on both radiative and conductive heat transfer information, while phase is less susceptible to, or not affected by, emissivity variations as being the ratio of quadrature and in-phase signal channels, both of which depend linearly on emissivity. As a consequence, as the lesion progresses, the phase channel data and the resulting 3D image reconstructions become considerably more valuable.

While lacking the 3D reconstruction capability of eTC-PCT, the SF-TWR (and by extension LIT) technique provides higher levels of SNR and contrast where 2D images are concerned. As mentioned, the primary reason for the higher SNR of SF-TWR phase output compared with eTC-PCT is the fact that SF-TWR employs square-wave laser illumination (i.e., 50% duty cycle), which is the maximum possible thermal-wave amplitude condition, compared with eTC-PCT’s use of five 40-ms pulses in the span of 12 s, a much lower duty cycle. The second contributing factor is that SF-TWR calculates its output by integrating the thermal relaxation signal received from the full duration of the measurement, thus presenting a depth-integrated result, whereas eTC-PCT applies a time-slicing algorithm to the CC result to enable 3D output and depth-resolved 2D slices, as used in Figs. 5 and 6 (each image is the first slice from each experiment). Additionally, while the SF-TWR system power was set at ∼100% MPE in this study, the eTC-PCT system energy level was set at 65% MPE. Therefore, eTC-PCT has considerably more capacity for using higher energy levels, which would result in increased SNR.

Other than the relative power levels and SNR values, the primary differentiating factor between the two modalities is their ability to visualize the progression of the demineralization. In this regard, the tomographic capability of eTC-PCT enables the visual representation of the lesion depth through depth-resolved 3D reconstructions. Although SF-TWR has higher SNR, by comparing the results it can be seen that eTC-PCT features more consistent trends, resulting in a better correlation between SNR, variance, and caries progression, which in turn translates to higher sensitivity and reliability for monitoring the severity and progression of demineralization.

The amplitude- and phase-based outputs of the eTC-PCT modality can potentially be employed in detecting and monitoring early caries with a low detection threshold and high sensitivity to change. It must be noted, however, that, due to the diffusive nature of the thermophotonic imaging mechanism, eTC-PCT cannot be easily used to evaluate the lesion depth quantitatively. Very recently, a unique spatial-gradient-gate adaptive filter of MIR camera images from different subsurface depths was introduced successfully, revealing absorber true spatial extent from diffusive thermophotonic images and restoring pre-diffusion lateral image resolution beyond the Rayleigh criterion limit in diffusion-wave imaging science.^14^ Studies are underway to apply this method to eTC-PCT dental image depth determination.

**Fig. 14:**
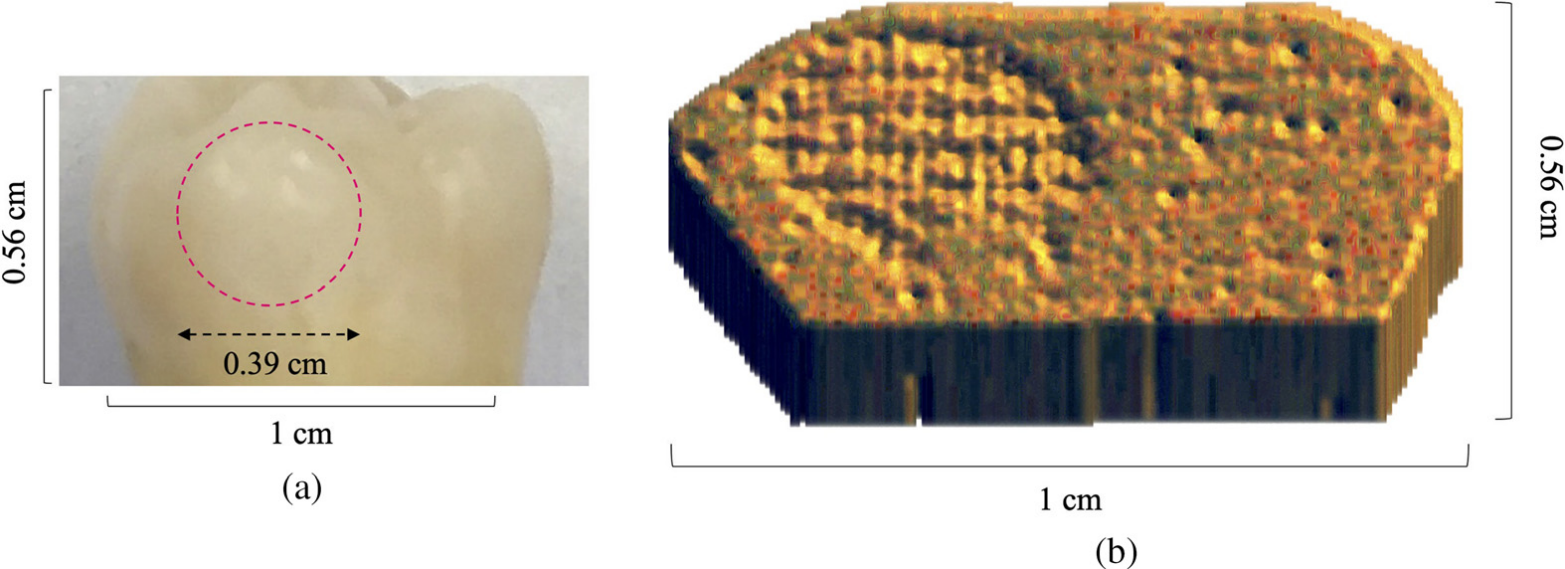
3D reconstruction of the erosion lesion on tooth E1. (a) The visible light image of E1, with the eroded treatment window outlined in a pink circle. (b) The 3D reconstruction of the surface erosion using the eTC-PCT amplitude delay channel data. The erosive depression on the tooth surface can be seen on the left side of the 3D reconstruction.

The erosion imaging results show that eTC-PCT has lower sensitivity to surface erosion compared with dental caries. This outcome is physically expected due to the fact that the primary contrast for imaging erosive removal of dental enamel material, particularly at early stages, is inherently lower than imaging dental caries because eTC-PCT signals are based on photon absorption in the residual substrate material that forms a thin layer. Therefore, image contrast is essentially due to secondary demineralization of the eroded residue, which only becomes more pronounced with increasing erosion, yet, remains much weaker than primary dental caries demineralization. As seen in Figs. 12 and 13, the eTC-PCT amplitude channel seems to be more suitable for imaging surface erosion compared with the phase channel. This is similar to very early caries, with the intensity of the conductive component of the photothermal signal that controls the phase lag being comparatively weaker than the radiative component. Nevertheless, eTC-PCT is capable of providing tomographic 3D reconstructions of the erosive lesion. Figure 14(b) presents the 3D reconstruction for sample E1 [Fig. 14(a)] after 2 min of erosion. As the phase channel data were highly noised, this reconstruction was generated using the eTC-PCT amplitude-delay data only. To calculate the amplitude-delay output, for each pixel of each slice, the time delay corresponding to the peak amplitude value gave the amplitude delay time value of that pixel. In this case, the higher absorption of the eroded region resulted in a near-zero delay time for the IR radiation amplitude peak emerging from this region, while the lower absorption of the surrounding region results in a longer delay time for the amplitude peak from those regions.

The near-zero value of the amplitude peak of the first (surface, t=0) eTC-PCT slices of the eroded region can be interpreted as a depression with a 3D depth profile compared with the surrounding region. This depression shows a textured bottom surface and becomes deeper as the erosion progresses, causing even higher absorption and thus more eTC-PCT slices with near-zero amplitude delay times for the eroded window. There is a precedent for this image behavior. As erosion results in a loss of surface material and surface etching, a depression/and or change in texture in the tooth surface is the effect observed using methods such as confocal laser scanning microscopy and surface profilometry.^26^^,^^27^

Based on these results, we demonstrated that the eTC-PCT amplitude channel is capable of detecting early-stage dental surface erosion. To our best knowledge, currently there is no clinically available dental imaging modality capable of detecting or visualizing this lesion type, including radiographs. The eroded surface was further examined with μCT, which was found incapable of revealing the eroded region. While the eTC-PCT 3D reconstructions do not provide quantitative depth information regarding the eroded region, these reconstructions can be employed as a useful and easy-to-interpret guide to monitor the progression of surface erosion severity as a valuable non-invasive companion to dental clinicians.

## 6 Conclusions

eTC-PCT shows significant promise as a potential non-invasive imaging modality in dental clinical practice, combining an invaluable host of features crucial to this application. This methodology has a low detection threshold and high sensitivity to progression of early dental caries and surface erosion and is able to detect lesions before they can be detected through visual inspection or x-ray radiography. Furthermore, it features no ionizing radiation, works well below the MPE, and is capable of 3D reconstructions of the extent of early subsurface dental lesions, making it suitable for early diagnosis and frequent monitoring of demineralization caries and enamel surface erosion. These traits can be valuable in preventive dentistry and may guide the use of remineralization treatments in place of invasive fillings.

While depth-integrated methods such as LIT and SF-TWR might be capable of higher SNR in dental imaging, they lack eTC-PCT’s sensitive carious lesion and erosion progression monitoring and 3D imaging capabilities. However, as all three modalities can be employed on a single instrumental setup, they can be used in tandem to complement each other for an optimized multi-modal thermophotonic dental diagnostic imaging clinical program.

## 7 Appendix: Comparing LIT with SF-TWR

LIT is the oldest and most established photothermal imaging method; it has been applied to dental imaging for detection of early caries in multiple studies.^9^^,^^10^^,^^28^ The recently developed SF-TWR^18^ operates on the same physical principles as LIT and provides similar output, but it employs a different signal processing algorithm based on the thermal wave radar concept, which can result in images with higher SNR compared with LIT.^29^ In SF-TWR, the starting and ending frequencies of an LFM chirp are the same, making it directly comparable to (and technically an evolution of) LIT/TPLI. SF-TWR uses the same hardware but has been shown to achieve higher SNR over a considerably wider range of modulation frequencies irrespective of camera frame-rate limitations.^18^ This feature was tested in dental caries imaging applications in this study.

**Fig. 15:**
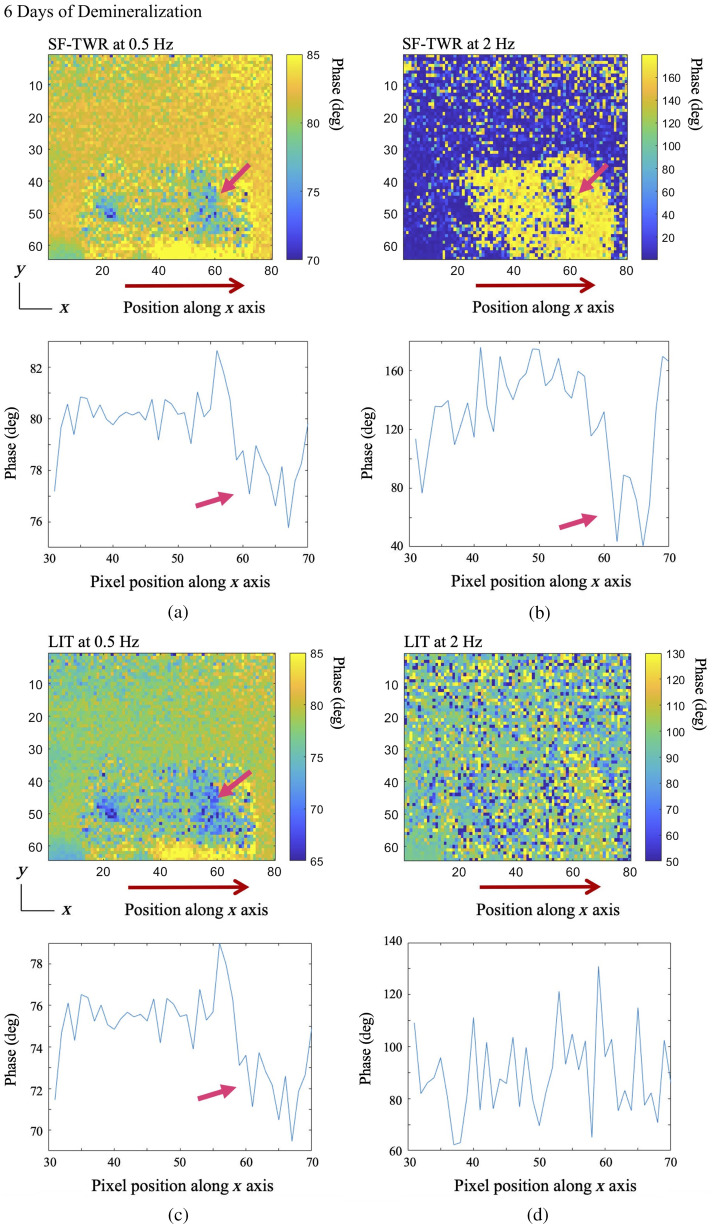
Comparison of SF-TWR and LIT outputs for imaging early artificially generated dental caries at 6 days of artificial demineralization on sample D1. Below each image, a corresponding pixel intensity plot is provided. The plots average the intensity of the pixels located on the tooth along the x-axis positions 30 to 70, marked by the red arrow below each image. The pink arrows mark the demineralized treatment window in each image/plot. It is observed that, while LIT and SF-TWR provide similar results at 0.5 Hz, SF-TWR provides higher SNR and detail at a higher frequency (2 Hz). Dimensions for all images: W=1.25  cm (±0.1), H=1  cm (±0.1).

**Fig. 16:**
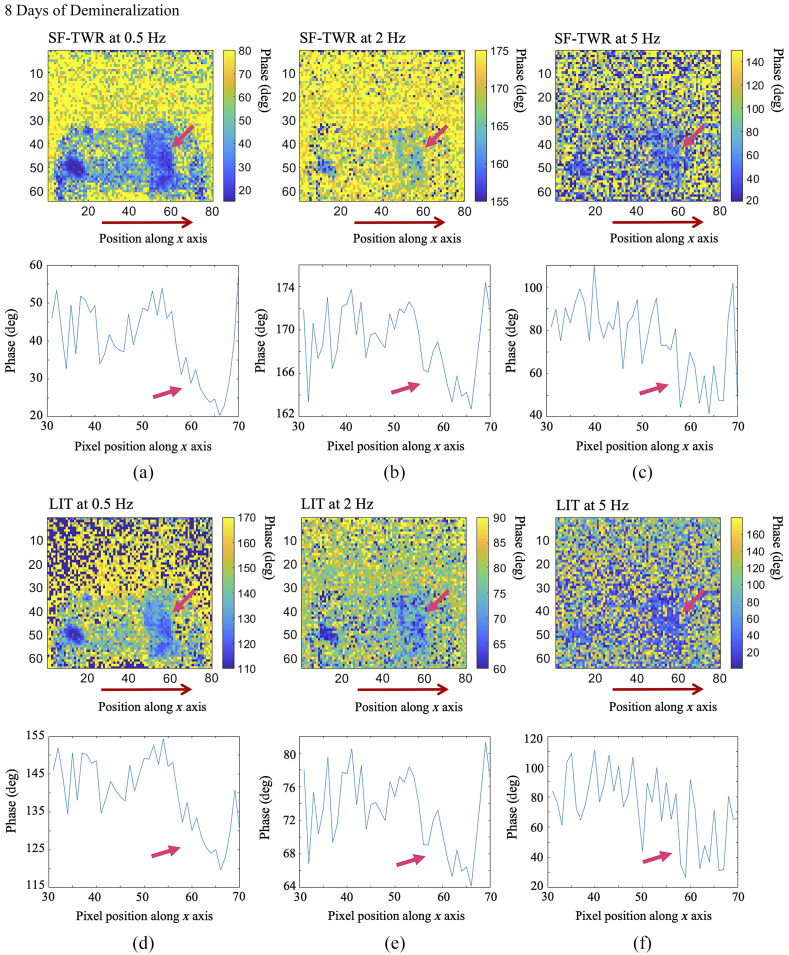
Comparison of SF-TWR and LIT outputs for imaging early artificially generated dental caries at 8 days of artificial demineralization on sample D1. Below each image, a corresponding pixel intensity plot is provided. The plots average the intensity of the pixels located on the tooth along the x-axis positions 30 to 70, marked by the red arrow below each image. The pink arrows mark the demineralized treatment window in each image/plot. Similar to Fig. 15, it is observed that, while LIT and SF-TWR provide similar results at lower frequencies, SF-TWR provides higher SNR and detail as frequency is increased. Dimensions for all images: W=1.25  cm (±0.1), H=1  cm (±0.1).

Given that one of the primary considerations for any non-invasive dental imaging modality has been the clinical practicality of the method and its compliance with the MPE limitations, for this comparative study, LIT and SF-TWR power levels were restricted to ∼100% MPE ($∼0.3\;W/{\mathrm{cm}}^{2}$). LIT imaging was carried out on one of the selected tooth samples used in the SF-TWR and eTC-PCT early caries studies presented in this paper (sample D1) and employed the same IR camera and laser. At 6 and 8 days of demineralization, LIT was performed on the same dental imaging data used for SF-TWR (i.e., both processing methods were applied to the same collected and stored data set) at several modulation frequencies (0.5, 2, and ≥5  Hz). At the selected power level, for frequencies >5  Hz, both methods yielded highly noised results and did not provide very useful contrast from teeth. Figures 15 and 16 present the phase results for the comparison of SF-TWR and LIT. The results show that, while LIT and SF-TWR have identical outputs below 1 Hz, as modulation frequency increases SF-TWR consistently exhibits more detailed images, higher SNR, or both.

The pixel intensity plots below each of the images in Figs. 15 and 16 are calculated by averaging the pixel phase values on the tooth on positions along the red arrows, which mark the location of the demineralized treatment window. It must be noted that the similarity of the results at 0.5 Hz is also evident from the pixel intensity plots accompanying each image: the LIT and SF-TWR images have different phase range values as they are processed with different phase reconstruction formulas,^18^ leading to different color contrast; however, the phase difference source data are the same for both. After 6 days of demineralization (Fig. 15), SF-TWR is capable of providing contrast data from the lesion at 2 Hz, while the LIT signal becomes highly noised, with no discernible data present either in the output image or the pixel intensity plot. At 8 days of demineralization (Fig. 16), both methods provide similar results up to 2 Hz, and both still provide some contrast data at 5 Hz; however, SF-TWR exhibits higher SNR for the treatment window, as seen in the pixel intensity plots. The results show that at the chosen excitation power level, the SF-TWR modality is capable of providing measurable contrast data for a wider range of modulation frequencies when imaging lesions subjected to shorter demineralization durations that exhibit weaker photothermal signals.

Based on this comparison between the two dynamic thermographic methods, SF-TWR was chosen to be compared with the eTC-PCT system (Sec. 3). In conclusion, it was found that SF-TWR can exhibit higher SNR while eTC-PCT is capable of higher sensitivity to lesion change and 3D reconstruction, both of which are essential for monitoring lesion progression. It is important to note that both imaging modalities can be implemented on the same instrumentation system and used in a complementary manner to facilitate the dental clinical diagnosis of early demineralization caries and erosion.
